# Voices of endocrinology: contemporary insights in endocrine and metabolic disorders

**DOI:** 10.20945/2359-4292-2026-0001

**Published:** 2025-12-08

**Authors:** Cesar Luiz Boguszewski

**Affiliations:** 1 Serviço de Endocrinologia e Metabologia, Departamento de Clínica Médica, Universidade Federal do Paraná (SEMPR), Curitiba, Brasil; 2 Editor-in-Chief Archives of Endocrinology and Metabolism, São Paulo, SP, Brasil

This special issue of the *Archives of Endocrinology and Metabolism* (AE&M) brings together invited narrative reviews written by experts from around the world. “*Voices of Endocrinology: contemporary insights in endocrine and metabolic disorders*” highlights current advances, ongoing controversies, and emerging directions across endocrine science and clinical practice. Each article reflects both the state of the art and the personal perspectives of distinguished leaders who have contributed over the years to shaping our understanding of the topics addressed in this issue.

Manuel Aguiar-Oliveira and cols. describe the emerging field of dermatoendocrinology and discuss the clinical diagnosis of various cutaneous manifestations associated with endocrine disorders (^1^). Likewise, Svenja Leibnitz, Cihan Atila and Mirjam Christ-Crain from the University of Basel, Switzerland, explore novel concepts related to oxytocin, emphasizing its potential for future therapeutic applications (^2^).

Coagulation disorders and the increased risk of thromboembolic events in patients with Cushing’s syndrome have been widely debated in recent years. In their article, Amit Akirov and Maria Fleseriu revisit current recommendations on anticoagulation therapy in this group of patients, addressing key gaps that hinder the implementation and standardization of protocols across different clinical settings (^3^).

The group led by Richard Quinton synthesizes the key elements of three recently published guidelines on female hypogonadism, providing a timely and practical update for clinicians managing this condition (^4^). Meanwhile, pediatric endocrinologists will benefit from the comprehensive review by Rodolfo Rey and his co-authors from Argentina, which offers an updated clinical approach to delayed puberty in males (^5^). At the opposite end of the age spectrum, Andrea Giustina and his group from Italy examine the role of vitamin D in preventing bone, muscle and adipose deterioration in the elderly, while Sergio Setsuo Maeda and his colleagues from the Federal University of Sao Paulo present an overview of the roles of micronutrients in bone health (^6^,^7^).

Multidisciplinary perspectives spanning bench-to-bedside approaches in the management of obesity, diabetes and metabolic disorders, are also featured in this special issue. Isabela Busto Silva and Manel Puig-Domingo review emerging mechanisms and current understanding of the thyroid-gut axis, highlighting its potential clinical implications (^8^). The pervasive nature of weight stigma in society and among healthcare professionals is covered in the review by Stuart Flint, Paula V. Sozza and Adrian Brown, who present and discuss a whole intervention framework aimed at reducing the impact of stigma on individuals living with obesity (^9^). Ana Flavia Moura and Roberto Pecoits-Filho provide a nephrology-oriented view on the cardio-kidney-metabolic framework, emphasizing the bidirectional relationship between kidney disease and cardiometabolic events and underscoring the need for enhanced multidisciplinary coordination (^10^). In a similar vein, the international team led by Anand Vaidya from Boston, USA, present novel evidence from pathology, biochemistry, proteomic and genetic studies, implicating subclinical primary aldosteronism in the pathogenesis of cardiorenal disease (^11^). Finally, the wide range of manifestations of diabetic autonomic neuropathy - one of the most serious and often under-recognized complications of diabetes - is addressed by Lucianne Tannus and Roberta Cobas in their review, which integrates basic, epidemiological, and clinical data to provide a comprehensive understanding of this condition (^12^).

Together, the articles of this special edition of AE&M provide clinicians, researchers, trainees and healthcare providers with a rich and diverse set of perspectives to guide the understanding and management of complex endocrine and metabolic disorders in an era of rapid scientific and clinical advancement.

## Data Availability

datasets related to this article will be available upon request to the corresponding author.
